# A brief and efficient stimulus set to create the inverted U-shaped relationship between rhythmic complexity and the sensation of groove

**DOI:** 10.1371/journal.pone.0266902

**Published:** 2022-05-19

**Authors:** Jan Stupacher, Markus Wrede, Peter Vuust

**Affiliations:** 1 Department of Clinical Medicine, Center for Music in the Brain, Aarhus University & The Royal Academy of Music Aarhus, Aalborg, Denmark; 2 Institute of Psychology, University of Graz, Graz, Austria; 3 Department of Clinical Medicine, Aarhus University, Aarhus, Denmark; University of Western Ontario, CANADA

## Abstract

When listening to music, we often feel a strong desire to move our body in relation to the pulse of the rhythm. In music psychology, this desire to move is described by the term *groove*. Previous research suggests that the sensation of groove is strongest when a rhythm is moderately complex, i.e., when the rhythm hits the sweet spot between being too simple to be engaging and too complex to be interpretable. This means that the relationship between rhythmic complexity and the sensation of groove can be described by an inverted U-shape (Matthews 2019). Here, we recreate this inverted U-shape with a stimulus set that was reduced from 54 to only nine rhythms. Thereby, we provide an efficient toolkit for future studies to induce and measure different levels of groove sensations. Pleasure and movement induction in relation to rhythmic complexity are emerging topics in music cognition and neuroscience. Investigating the sensation of groove is important for understanding the neurophysiological mechanisms underlying motor timing and reward processes in the general population, and in patients with conditions such as Parkinson’s disease, Huntington’s disease and motor impairment after stroke. The experimental manipulation of groove also provides new approaches for research on social bonding in interpersonal movement interactions that feature music. Our brief stimulus set facilitates future research on these topics by enabling the creation of efficient and concise paradigms.

## Introduction

In music psychology, the experience of groove is often defined as a pleasurable state of wanting to move one’s body in relation to the pulse of a musical rhythm [1–4]. Recent findings suggest that we feel a strong desire to move our bodies when listening to music with a moderate amount of *rhythmic complexity*, whereas in comparison, low and high amounts of *rhythmic complexity* decrease our desire to move [5, 6]. Consequently, the relationship between *rhythmic complexity* and the sensation of groove can be described by an inverted U-shape. Besides *rhythmic complexity*, Matthews and colleagues [5] investigated the influence of *harmonic complexity* on the sensation of groove and found that wanting to move ratings were similar for low and moderately complex harmonies, but dropped for a highly complex harmony. The present study tests whether these effects of *rhythmic* and *harmonic complexity* can be replicated with a subset of nine stimuli from the original set of 54 stimuli used by Matthews and colleagues [5].

Rhythmic complexity, and the related affective and behavioral responses, play important roles in recent research on music-supported movement therapies, social bonding, and neurophysiological mechanisms underlying motor timing and reward processes. Predicting how music unfolds and develops over time is a rewarding, pleasurable process [7, 8] that involves neural auditory-motor interactions [9, 10]. With music that is rated as high-groove, neural auditory-motor interactions are more affected than with low-groove music [4]. Music that facilitates the sensation of groove might therefore be especially effective in therapeutic programs for improving body movements in individuals with conditions such as Parkinson’s disease, Huntington’s disease and motor impairment after stroke [11, 12]. Importantly, in patients with motor impairments, the most groove-inducing rhythms might be shifted from moderate to low rhythmic complexities when compared with healthy control participants [13]. A similar shift of the inverted U-shape towards simpler rhythms could also occur for cochlear implant users—but research on this topic is lacking.

Rhythmic complexity is also important for social bonding in interpersonal movement interactions that feature music. A clear perception of the temporal structure of music is crucial for providing a meaningful social context in which the movements of oneself and others can be interpreted and evaluated. Interestingly, recent findings suggest that a moderate level of rhythmic complexity may be favorable for social bonding when moving together with music: The feeling of social closeness tends to follow an inverted U-shape in relation to rhythmic complexity [14]. Although the inverted U-shape of rhythmic complexity can also be found in social interactions, it remains an open question whether and how groove experiences differ in individual and social contexts.

Combined with neuroimaging, groove research can help us understand the role of motor and reward networks when making and listening to music [4, 15, 16]. In neuroimaging, but also in behavioral studies, one of the outstanding questions is how rhythmic and harmonic complexity contribute to motor behavior, reward and pleasure. To facilitate future research on all of the previously mentioned topics, we present a brief stimulus set of nine rhythms that reproduces the effects of rhythmic and harmonic complexity found in an experiment by Matthews and colleagues [5] with a stimulus set of 54 rhythms.

## Method

### Participants

Data were collected in an online questionnaire completed by 174 participants. Five participants reported a history of motor-related neurological diseases and were excluded. The resulting 169 participants (116 female, 53 male) had a mean age of 25.1 years (*SD* = 6.1). One hundred fifty-one participants were Danish; the rest of the participants came from 13 different countries (Austria [n = 1], Belgium [1], Canada [1], France [2], Germany [3], Iceland [1], Lithuania [1], Mexico [1], Norway [2], South Korea [1], Sweden, UK [2], and Uruguay [1]). Twenty-five participants reported playing an instrument and singing, 75 participants reported playing an instrument but not singing, 14 participants reported singing but not playing an instrument, and 55 participants reported not playing an instrument and not singing. The sample size was comparable to Matthews and colleagues’ [5] sample of 201 participants. The study was conducted in accordance with the guidelines from the Declaration of Helsinki and the Danish Code of Conduct for Research Integrity and Aarhus University’s Policy for research integrity, freedom of research and responsible conduct of research. All collected data included no personally identifiable information.

### Stimuli

**Fig 1:**
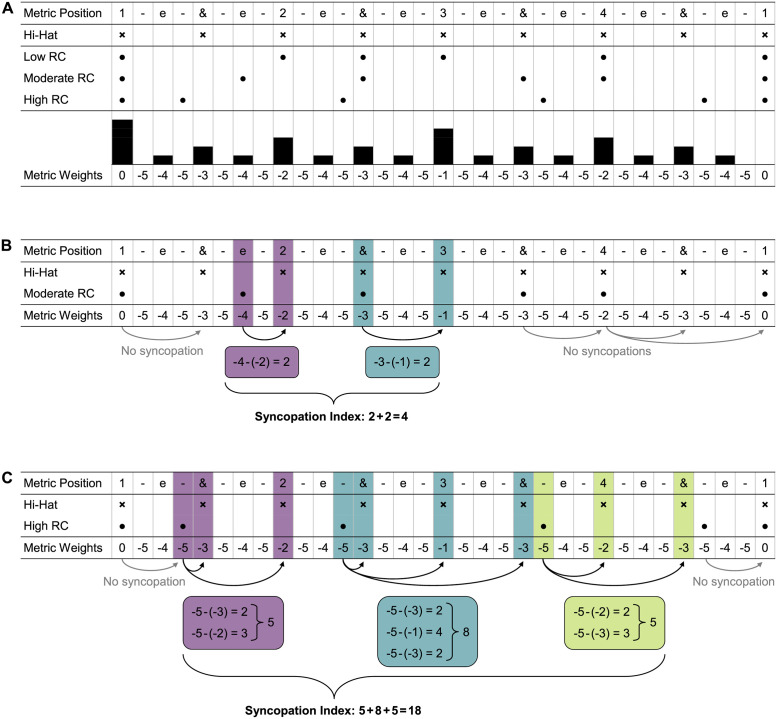
Description of the rhythms with metric weights and syncopation measures. A) Low, moderate and high rhythmic complexity (RC) stimuli. Each onset is represented by a dot (●) with the corresponding metric weight noted below. The eight-note hi-hat (×) is part of each of the three stimuli. B and C) Calculations of the syncopation index following Fitch and Rosenfeld (2007) and Matthews et al. (2019) for the moderate and high rhythmic complexity stimuli. A syncopation occurs when a rest (here at the eight-note level) is preceded by the onset of a note of lesser weight. The summed up differences between syncopated notes and rests make the syncopation index.

Nine audio clips were selected based on subjective musicality by author MW from a set of 54 stimuli used by Matthews and colleagues [5]. The audio clips were ten seconds long and consisted of rhythmic patterns played with one chord in a piano timbre. Every rhythmic pattern consisted of five onsets per bar, which were repeated for four bars in total. The *rhythmic* and *harmonic complexity* of the patterns had three levels. *Rhythmic complexity* varied between low, moderate and high levels of syncopation (Fig 1A). The chords were in D major and varied between low *harmonic complexity* (D major triad and two inversions), moderate *harmonic complexity* (four note chords with extensions), and high *harmonic complexity* (flat ninth interval between chord note and extension). For more information about the stimuli, see Matthews et al. [5]. Roughness was calculated with the MIR-toolbox [17] to estimate *harmonic complexity* of the selected stimuli. Pulse clarity (MIR toolbox) and syncopation index [5, 18] were calculated to estimate *rhythmic complexity*. Mean roughness was lowest for low *harmonic complexity* (188), followed by moderate (213), and high (266) *harmonic complexity*. Pulse clarity (PC) was highest and syncopation index (SI) was lowest for low *rhythmic complexity* (PC: 0.72, SI: 0), followed by moderate (PC: 0.31, SI: 4), and high (PC: 0.28, SI: 18) *rhythmic complexity*. For details on the calculations of the syncopation indices, see Fig 1B and 1C. The nine stimuli can be found online at https://researchbox.org/487.

### Procedure

Each audio clip was presented once in randomized order. After each stimulus, participants rated the groove of the audio clip on a continuous slider ranging from “not groovy” on the left to “very groovy” on the right. The numerical values ranged from 1 on the left side to 101 on the right side, but participants could not see these values. Groove was defined as “wanting to move in time with the music” [1, 2, 4]. In contrast to Matthews et al. [5] who asked, “How much does this musical pattern make you want to move?” the present study directly mentioned the term groove and connected it to the urge to move with a musical rhythm.

## Results and discussion

**Fig 2:**
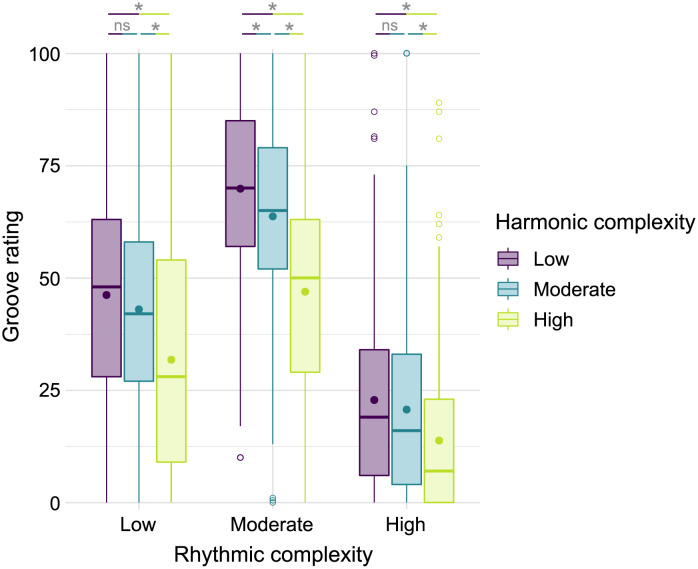
Groove ratings as a function of rhythmic and harmonic complexity. Dots represent mean groove ratings. Boxplots: The centerline represents the median. The lower and upper ends of the boxes correspond to the first and third quartiles. Whiskers represent lowest and highest values within 1.5 × interquartile range (IQR) from the lower and upper quartiles, respectively. Circles represent values outside 1.5 × IQR. *ns = nonsignificant*, * p < *.001*.

In line with previous research by Matthews et al. [5] and Witek et al. [6], groove ratings followed an inverted U-shape when plotted against *rhythmic complexity*, i.e. syncopation (Fig 2). The strongest sensation of groove was reported for patterns with a moderate amount of *rhythmic complexity* (*M* = 61.2, *SD* = 23.7), followed by low (*M* = 41.3, *SD* = 24.4) and high (*M* = 20.1, *SD* = 20.5) *rhythmic complexity*. The manipulation of *harmonic complexity* also led to similar results as in Matthews et al. (2019): Groove ratings were highest for low *harmonic complexity* (*M* = 47.3, *SD* = 29.0) followed by moderate (*M* = 43.5, *SD* = 27.4) and high (*M* = 31.8, *SD* = 26.4) *harmonic complexity*.

An ANOVA on groove ratings with the within-subject factors *rhythmic complexity* and *harmonic complexity* revealed main effects of *rhythmic complexity* (*F*(2,1344) = 686.30, *p* < .001, *η*^2^ = .46) and *harmonic complexity* (*F*(2,1344) = 105.43, *p* < .001, *η*^2^ = .07) as well as an interaction between the two factors (*F*(4,1344) = 7.05, *p* < .001, *η*^2^ = .01). With the large effect size of *η*^2^ = .46, the factor *rhythmic complexity* may be more important for the sensation of groove than *harmonic complexity*, with a much smaller effect size of *η*^2^ = .07. This finding is in accordance with Matthews et al. [5], who suggest that “rhythm plays a primary role in generating the sensation of groove, with harmony providing a modulatory role through its effect on pleasure”. It is important to note that the inverted U-shape of *rhythmic complexity* might be more prominent in a sample of professional musicians and less prominent in non-musicians in comparison to our sample of mixed musical expertise [5].

The interaction between the two factors was unpacked with Bonferroni-corrected paired t-tests (see Fig 2). Differences in groove ratings between low and moderate *harmonic complexity* were nonsignificant in stimuli with low and high *rhythmic complexity*, but significant in stimuli with moderate *rhythmic complexity*. All other pairwise comparisons between *harmonic complexity* levels within each *rhythmic complexity* level were significant (p < .001). Data of the individual participants can be found in S1 Table. In accordance with findings of Matthews et al. [5], our study provides further evidence for the assumption of an inverted U-shape relationship between rhythmic complexity and the sensation of groove. This inverted U-shape can be explained using the theory of predictive coding of music, in which it is proposed that music perception and action are shaped by bottom-up sensory input on one hand, and top-down predictive brain models on the other hand [19, 20]. According to the predictive coding of rhythm [20], a listener compares an internally generated predictive model with the actual sensory input. Music with moderate rhythmic complexity is regular enough for a listener to create a predictive mental model of beat and meter that is possible to move in time to, whereas the syncopations within the rhythm result in prediction errors. These prediction errors can be reduced either by updating the model to better align with the sensory input, or by changing the input, for example by moving one’s body in time with the beat to add a proprioceptive dimension. In these ways, reducing prediction errors increases top-down engagement, embodiment, pleasure, and reward. If the rhythms are very simple, however, the predictions and the input match almost perfectly, leading to few prediction errors, and therefore fewer updates of the predictive model and less top-down engagement. If the rhythms are very complex, the listener is unable to create an appropriate predictive model at all, precluding the possibility of updating the model. Compared to moderately complex rhythms, both very simple and very complex rhythms may therefore reduce top-down engagement, pleasure, and embodied experiences associated with updating and maintaining the predictive model.

Syncopation is one of the most discussed musical features related to groove [5, 6, 16, 21–23], but it is of course not the only one. Other rhythmic and sonic features connected to the sensation of groove are energy in bass frequencies [24, 25], event density [22, 26] cf. [25], beat salience / pulse clarity [26] cf. [22, 25], and tempo [1] cf. [22] cf. [27]. Microtiming—intended and expressive onset deviations from the metric grid in the range of a few tens of milliseconds—is another often-discussed feature of groove. However, empirical studies come to contradictory conclusions with positive, negative, and null effects of microtiming deviations on groove ratings (see e.g., [28] for an overview). The brief stimulus set presented here can be used to investigate how syncopation interacts with the above-mentioned features in evoking the sensation of groove.

As the current musical stimuli are synthesized, their (micro)rhythmic and sonic features can be easily adapted for future experiments. Their synthesized nature also minimizes potential effects of familiarity that can occur with popular music [29]. However, the high control and internal validity associated with synthesized stimuli, a fully crossed randomized design, and individual data collection come with a decrease in ecological validity. Future experiments could therefore build on the recent findings by comparing synthesized versus performed versions of the stimulus set, and individual versus collective listening environments.

The application-oriented conclusion of the present study is that the inverted U-shaped relationship between *rhythmic complexity* and the sensation of groove can be shown efficiently with a set of nine stimuli that are only rated once. The effects of *harmonic complexity* on groove ratings measured with this small stimulus set are also similar to the effects of the original study with 54 stimuli [5]. This is especially important for future studies on rhythm perception and the sensation of groove with time constraints and complex designs. The brief stimulus set facilitates efficient combinations of groove paradigms and clinical applications, such as Parkinson’s, Huntington’s, and stroke therapies, or cochlear implant research. Participants in most of these groups particularly profit from short paradigms. Furthermore, the stimulus set’s conciseness is ideal for large-scale online experiments comparing participants who, for example, differ in cultural background, musical expertise, dance training, motor skills, empathy, or age. These types of groove research paradigms will help us to better understand the interplay between timing processes, movement, social behavior, and pleasure in music listening, music making, and dance.

## Supporting information

S1 Table Individual groove ratings of participants. The table shows all combinations of low, moderate, and high rhythmic complexities (LR, MR, and HR, respectively) with low, moderate, and high harmonic complexities (LH, MH, and HH, respectively). Ratings were given on a continuous scale from 1 on the left to 101 on the right. Participants could not see these values.
(PDF)
