# Supplementary material for: A brief and efficient stimulus set to create the inverted U-shaped relationship between rhythmic complexity and the sensation of groove
Source: PLoS One. 2022 May 19;17(5):e0266902. doi: 10.1371/journal.pone.0266902 (PMC9119456; doi:10.1371/journal.pone.0266902)
Supplement: S1 Table — The table shows all combinations of low, moderate, and high rhythmic complexities (LR, MR, and HR, respectively) with low, moderate, and high harmonic complexities (LH, MH, and HH, respectively). Ratings were given on a continuous scale from 1 on the left to 101 on the right. Participants could not see these values. (PDF) [file pone.0266902.s001.pdf]

Supporting Information

A Brief and Efficient Stimulus Set to Create the Inverted U-Shaped Relationship between Rhythmic Complexity and the Sensation of Groove

Jan Stupacher [1,2], Markus Wrede [3], Peter Vuust [1]

[1] Center for Music in the Brain, Department of Clinical Medicine, Aarhus University & The Royal Academy of Music Aarhus/Aalborg, Denmark

[2] Institute of Psychology, University of Graz, Austria

[3] Department of Clinical Medicine, Aarhus University, Denmark

Corresponding author:

Jan Stupacher
Center for Music in the Brain
Universitetsbyen 3, Building 1710
8000 Aarhus C, Denmark
ORCID: 0000-0002-2179-2508
Email: stupacher@clin.au.dk

S1 Table. Individual groove ratings of participants. The table shows all combinations of low, moderate, and high rhythmic complexities (LR, MR, and HR, respectively) with low, moderate, and high harmonic complexities (LH, MH, and HH, respectively). Ratings were given on a continuous scale from 1 on the left to 101 on the right. Participants could not see these values.

Participant	LR_LH	LR_MH	LR_HH	MR_LH	MR_MH	MR_HH	HR_LH	HR_MH	HR_HH	Singing	Instrument
1	10	17	15	60	82	69	1	6	6	no	yes
2	64	62	56	64	57	54	32	41	32	no	no
3	1	1	1	101	87	1	1	1	1	no	yes
4	101	76	80	101	101	101	101	69	53	no	yes
5	40	47	21	50	48	53	1	9	1	no	no
6	41	42	27	101	74	52	10	18	1	no	yes
7	13	1	1	88	74	76	1	1	24	no	no
8	84	67	63	95	67	71	27	28	4	no	yes
9	9	1	22	73	46	22	1	1	1	no	no
10	48	40	40	52	51	51	14	11	19	yes	yes
11	62	73	72	89	82	59	9	19	4	no	yes
12	58	21	40	56	65	50	5	8	1	yes	yes
13	52	23	65	51	87	30	9	7	3	no	yes
14	85	94	80	80	69	85	1	7	1	no	yes
15	72	59	1	101	1	33	1	1	1	no	yes
16	71	70	71	64	82	40	29	61	18	yes	no
17	47	67	58	60	55	54	37	39	38	no	no
18	21	73	37	79	84	84	24	7	7	no	yes
19	75	26	1	65	58	1	1	1	1	yes	no
20	52	44	40	78	75	74	25	12	16	no	yes
21	18	13	9	89	62	68	51	9	26	no	yes
22	40	40	20	71	65	64	9	13	8	yes	yes
23	13	16	2	64	64	98	12	21	1	no	no
24	69	63	61	93	56	57	44	45	39	no	yes
25	63	64	26	97	101	17	61	42	1	yes	yes
26	68	51	1	85	88	61	1	8	1	no	yes
27	23	32	9	80	46	43	43	28	25	no	no
28	10	21	8	50	51	54	12	48	48	yes	yes
29	8	15	9	53	42	47	1	1	12	no	yes
30	49	60	53	50	59	58	49	56	53	no	no
31	57	52	6	101	76	94	19	2	1	no	yes
32	73	58	6	42	71	2	82	74	88	no	yes
33	71	73	14	101	80	36	74	52	36	no	yes
34	21	30	16	60	39	31	8	17	9	no	yes
35	36	34	36	54	48	38	44	50	20	yes	no
36	69	62	71	87	77	75	56	62	58	no	no
37	53	64	58	62	95	70	11	46	4	no	yes
38	1	71	22	101	81	74	10	1	1	no	yes
39	92	27	44	78	71	34	59	60	16	no	no
40	61	48	5	35	75	40	8	37	2	no	yes
41	59	47	22	67	62	44	17	12	1	no	yes
42	82	58	65	78	89	58	25	19	19	no	no
43	51	35	60	81	72	22	36	37	10	no	no
44	42	45	24	84	51	30	33	12	1	no	no
45	51	53	44	56	53	21	48	47	8	no	yes
46	57	41	35	93	88	59	9	32	4	yes	yes
47	63	41	40	77	57	33	1	1	1	yes	yes
48	41	28	1	80	72	56	32	8	1	no	yes
49	53	58	61	88	78	77	36	26	29	yes	yes
50	62	23	7	92	91	18	43	23	9	no	yes
51	5	9	1	11	23	3	1	1	1	no	no
52	24	16	1	95	81	31	18	1	1	yes	yes
53	66	101	41	73	101	72	32	1	1	no	yes
54	59	55	54	68	87	64	56	64	37	no	yes

55	31	32	53	90	67	55	22	1	10	yes	yes
56	62	59	58	86	87	80	32	47	43	no	no
57	72	46	49	71	77	55	52	13	4	no	no
58	15	36	13	45	43	58	20	25	5	no	yes
59	65	34	14	66	52	53	47	41	63	no	yes
60	24	21	20	50	47	46	51	51	34	no	no
61	23	26	26	90	71	36	9	10	34	yes	yes
62	49	46	34	70	63	47	15	22	9	no	yes
63	66	71	72	93	85	87	1	37	28	no	no
64	93	91	60	101	101	66	68	76	58	no	yes
65	22	39	33	88	71	47	5	28	9	yes	yes
66	43	38	34	83	66	60	13	20	16	no	yes
67	54	51	28	84	58	63	56	51	7	yes	no
68	63	59	63	84	76	78	82	73	82	yes	yes
69	44	46	35	75	46	60	20	9	3	yes	yes
70	53	56	63	66	54	52	24	16	24	yes	yes
71	36	47	1	70	64	28	25	10	16	no	yes
72	46	31	45	53	68	50	13	3	1	yes	yes
73	48	47	55	66	74	53	48	34	12	yes	yes
74	11	10	8	19	14	10	1	1	1	no	no
75	30	25	8	94	67	37	21	23	9	no	yes
76	70	83	82	86	81	71	65	65	60	no	yes
77	38	39	6	64	77	64	22	34	14	no	yes
78	32	47	1	73	64	18	5	21	7	no	yes
79	54	49	1	57	60	42	101	24	1	no	no
80	53	53	52	91	87	67	10	22	1	yes	no
81	47	36	3	67	57	65	41	8	9	no	yes
82	2	2	3	53	2	7	56	1	2	no	no
83	101	68	88	87	87	90	1	1	7	no	yes
84	68	38	10	97	89	48	30	14	1	yes	yes
85	54	1	1	59	49	8	29	24	26	no	no
86	64	94	1	83	69	66	1	9	1	no	yes
87	32	33	36	49	48	45	13	17	1	yes	yes
88	93	66	39	85	80	73	25	19	12	yes	no
89	64	66	36	85	54	61	15	11	13	no	no
90	1	1	3	35	18	5	4	3	1	no	no
91	95	67	4	100	81	7	14	1	1	yes	yes
92	20	41	61	76	73	81	34	10	30	no	no
93	26	5	1	57	60	8	1	1	1	no	yes
94	34	39	55	62	54	61	18	11	35	no	yes
95	22	22	19	68	66	35	12	17	1	no	yes
96	40	55	28	58	56	56	20	21	1	no	no
97	57	28	19	90	69	73	41	1	1	no	yes
98	69	59	56	48	81	56	1	27	1	yes	no
99	49	50	20	67	70	64	48	25	23	no	yes
100	9	20	3	33	32	18	27	34	3	no	yes
101	56	60	61	59	59	66	40	22	29	no	no
102	28	55	1	77	53	12	24	9	1	no	no
103	97	71	73	89	101	77	1	1	1	no	no
104	62	53	59	74	75	62	59	35	43	yes	no
105	47	44	63	87	101	84	10	39	1	no	yes
106	49	36	36	68	77	41	27	3	6	no	yes
107	53	35	18	75	72	58	1	1	1	no	no
108	64	61	86	89	65	93	11	13	10	no	yes
109	97	80	33	71	63	36	9	1	25	no	yes
110	68	76	71	77	87	70	22	28	57	no	yes
111	37	36	30	55	41	46	26	12	9	yes	no
112	56	33	29	57	52	38	27	42	36	no	no
113	73	43	63	78	83	68	42	24	46	no	yes

114	27	48	58	62	53	46	25	16	1	yes	yes
115	20	31	20	43	45	43	27	29	30	no	no
116	30	26	14	37	42	17	30	14	19	no	yes
117	8	13	9	47	39	18	1	3	1	yes	yes
118	22	23	25	82	101	62	1	11	1	yes	yes
119	31	25	7	65	58	27	17	47	1	no	yes
120	22	32	1	30	61	9	1	1	1	no	yes
121	65	67	47	71	82	59	1	1	1	yes	yes
122	61	55	43	59	76	41	19	7	8	no	yes
123	57	19	15	74	69	46	21	1	2	no	yes
124	64	79	62	82	83	71	21	9	4	no	no
125	66	56	23	73	66	52	9	1	2	no	no
126	42	27	34	92	75	87	11	46	9	no	yes
127	7	14	10	45	45	27	3	4	4	no	yes
128	5	10	8	31	29	22	1	1	6	no	no
129	27	12	8	49	47	13	24	9	7	yes	no
130	66	63	45	93	70	57	11	20	12	no	yes
131	68	40	58	68	58	34	55	23	42	yes	no
132	45	44	22	45	59	24	28	41	16	no	yes
133	58	54	46	70	73	50	56	43	37	no	no
134	51	48	16	67	65	70	21	1	9	no	no
135	59	53	57	83	80	65	67	31	31	no	no
136	22	11	51	70	101	27	1	12	1	no	no
137	66	61	21	88	81	21	28	13	4	no	yes
138	10	1	7	33	29	1	2	1	1	no	no
139	36	31	27	69	48	16	25	9	21	no	no
140	56	81	14	69	28	61	35	60	8	yes	no
141	4	40	20	86	63	59	36	17	6	no	no
142	29	36	32	52	51	35	17	35	1	no	yes
143	64	41	16	79	23	64	32	19	9	yes	no
144	45	68	1	72	65	11	1	1	1	no	yes
145	50	50	34	59	59	35	37	25	25	no	yes
146	20	30	12	59	38	42	33	14	11	no	no
147	34	58	63	68	61	40	65	65	65	no	no
148	28	30	23	70	34	22	28	54	25	no	no
149	23	40	10	77	65	53	6	7	10	no	yes
150	79	73	9	18	1	13	6	1	1	yes	no
151	53	42	40	71	91	15	1	24	1	no	no
152	45	36	28	70	65	39	5	13	5	no	no
153	41	22	25	51	19	19	14	1	1	no	no
154	89	92	24	93	93	91	14	5	1	no	yes
155	63	67	64	101	101	92	8	36	28	no	yes
156	31	20	34	28	36	19	37	41	1	no	yes
157	70	75	85	100	96	7	16	19	14	no	yes
158	46	60	8	57	77	17	1	28	1	no	yes
159	75	18	66	85	46	31	7	2	8	no	no
160	33	28	19	98	33	96	1	4	23	no	no
161	46	36	33	66	53	24	2	1	1	no	no
162	35	48	36	55	25	52	13	29	26	no	no
163	79	79	68	80	83	79	9	25	18	no	yes
164	101	92	87	88	86	92	88	101	90	yes	yes
165	61	34	46	77	61	49	37	36	21	no	yes
166	1	38	101	99	101	1	1	1	1	no	no
167	43	55	31	46	66	63	11	50	34	no	no
168	16	25	29	55	67	50	1	1	1	no	yes
169	33	14	1	84	72	54	27	11	1	no	no
